# Evaluation of neuropsychological characteristics and attention bias in juvenile offenders, juvenile victims, and juveniles who have not participated in the criminal justice system

**DOI:** 10.3389/fpsyg.2023.1229044

**Published:** 2023-09-04

**Authors:** Büşra Patiz, Seda Bayraktar

**Affiliations:** ^1^Directorate of Judicial Support and Victim Services, Antalya Courthouse, Antalya, Türkiye; ^2^Department of Psychology, Akdeniz University, Antalya, Türkiye

**Keywords:** juvenile offender, juvenile victim, executive functions, attention bias, forensic neuropsychology

## Abstract

**Introduction:**

The increasing inclusion of children in the criminal justice system as “juvenile offenders” and “juvenile victims” has recently emerged as a severe and multifaceted problem. This study evaluates whether juvenile offenders differ from juveniles who have not participated in the criminal justice system and juvenile victims regarding executive function skills and attentional bias.

**Method:**

The participant group comprised 85 children aged 12–18, and the study setting was Turkey, utilizing one control group and two treatment groups with open criminal case files in Antalya Courthouse. The first treatment group consisted of 30 juvenile offenders; the second consisted of 30 juvenile victims. The control group consisted of 25 juveniles who were not juvenile offenders or victims. In this context, children’s executive functions were measured with the short-form Barratt Impulsivity Scale, the Raven Standard Progressive Matrices Test, the TBAG-form Stroop test, the Wisconsin Card Sorting Test, and the Istanbul 5 Cube Planning Test. Attentional bias was measured using a dot-probe task. Illiteracy, intellectual or developmental disability, and being a non-native Turkish speaker were the exclusion criteria for all three groups.

**Results:**

The study found that the scores of the juvenile offender group on the Barratt Impulsivity Scale were significantly higher than the children in the juvenile victim group and the children in the control group. For other tests measuring executive functions, the control group’s scores were significantly higher than juvenile offenders and juvenile victims. Regarding attentional bias, the children in the control group exhibited less attentional bias to negative stimuli than the juvenile offenders and victims.

**Discussion:**

Researchers have generally addressed the reasons that push children to crime and become victims of crime through individual, familial, and environmental reasons. However, the number of studies investigating the neuropsychological characteristics of children dragged into crime is relatively limited in our country. In addition, there is no study comparing the executive functions and attentional bias of children who are dragged into crime, victimized children, and children without a history of being dragged into crime and victimization. In this context, this study can highlight important implications for the judicial system regarding juvenile delinquency interventions.

## 1. Introduction

Like the Latin “crimen,” crime is defined in the literature as an action prohibited and punishable under the laws created by society or the state (Ögel, 2007). Due to its universality, crime is a concept explored, contemplated, and sought to be prevented by various societies and disciplines (Polat, 2009; Canter, 2010). Both field studies and meta-analyzes are at the forefront of today’s growing number of crime and neuropsychology research, emphasizing the importance of diverse cognitive functions in understanding the development and recurrence of crime and, consequently, victimization prevention (Mohr-Jensen and Steinhausen, 2016; Malarbi et al., 2017; Coenen et al., 2021). Understanding these cognitive functions will bring significant insights into various disciplines, including the judicial system, particularly regarding vulnerable criminal subjects, such as abused children or individuals compelled to commit crimes.

Laws and procedures specific to children have been established by legislators. These regulations aim to protect children’s involvement in or victimization from crime and reintegrate them into society. Within the scope of Turkey’s Child Protection Law no. 5395, the concept of a juvenile victim is addressed within the scope of children in need of protection, including both children dragged into crime (juvenile offender) and victimized children as children in need of protection (Child Protection Law, 2005). Examining the legal articles for children in various countries such as the United Kingdom, the United States, Canada, and Germany reveals that the goal is to protect and reintegrate children into society. This is reflected in the age of criminal responsibility, detention procedures, judicial proceedings, and sentence execution, all of which follow different rules than adults (Dünkel, 2002; Usc Ch. 403: Juvenile Delinquency, 2010; Minister of Justice and Attorney General of Canada, 2013; EU Countries, 2019).

Studies on the causes of juvenile delinquency have identified many factors that cause children to become involved in crime (Ögel, 2014). Examining these factors, determining the risks leading to juvenile incidences of crime, and delineating the protective factors against juvenile crime are essential to developing effective intervention methods for youths (Aksel and Yılmaz Irmak, 2014). Multiple approaches offer ways to classify risk factors and protective factors. Based on Bronfenbrenner’s (1977) Ecological Model, the most frequently used and accepted is the Multidimensional Psychosocial Approach. This approach states that all factors are interrelated and groups risk factors as individual, familial, and environmental.

One observes that overall, the literature focuses on adolescence as a period associated with children who become involved in crime (Brugman and Aleva, 2004; Güçlü and Akbaş, 2019, p. 323; Ögel, 2014) or display early-onset behavior problems (Shader, 2001; Fergusson et al., 2005). In addition, scholars have researched how the following relating to juvenile crime or victimization: psychiatric disorders (Pratt et al., 2002; Moore et al., 2013), family structure (Köknel, 2001; Thompson and Bynum, 2016), parenting characteristics (Loeber, 1990; Wright and Cullen, 2001), substance use (Assink et al., 2015; Belenko et al., 2017), social problems (Farrington and Welsh, 2008; Neto, 2009; Gönültaş and Kelebek, 2017), friendship relationships (Gül and Güneş, 2009; Susanu, 2019; Walters, 2019), and educational experiences (Maguin and Loeber, 1996; Cullen et al., 2008; Erbay and Gülüm, 2018).

Regarding child victimization, the literature has examples of research on how it relates to gender (Ullman and Filipas, 2005; Priebe, 2009; Fuller-Thomson and Agbeyaka, 2020), age (Finkelhor et al., 2005; Cammack and Hogue, 2017), family structure (Santrock, 2012; Mansbach-Kleinfeld et al., 2015; Straus and Smith, 2017), parental attitudes (Putnam, 2003; Giardino et al., 2018), friendship relationships (López et al., 2012; Hébert et al., 2017), and social problems (Doidge et al., 2017; Hinds and Giardino, 2017). Therefore, this study aims to closely and objectively assess whether there are differences between the neuropsychological characteristics of children dragged into crime or victimized children compared to the control group.

Previous studies have evaluated children’s executive functioning and attention bias in this context. Executive function skills enable a person to pay attention to any activity, think creatively and flexibly, manage emotions, control impulses, plan and initiate activities, self-evaluate performance, and remember and skillfully manage important information (Goldstein et al., 2014). As Miyake et al. (2000) suggested, executive functions are accepted as having a holistic model consisting of three components: inhibitory control, working memory, and cognitive flexibility (Diamond, 2013; Rosen et al., 2019). It has been stated that if individuals have healthy attention functions, they do not have problems with orientation, attention maintenance, or concentration. However, if attention functions are impaired, confusion in the mind and difficulty in concentration and attention maintenance can be observed. Due to its relationship with other cognitive processes, healthy attention functions emerge as an essential prerequisite for all other cognitive functions, especially memory (Mesulam, 2000).

In this context, attentional bias, which is another primary variable of the study, can be explained in three ways: directing attention to one stimulus, distracting attention away from one stimulus, and shifting attention from one stimulus to the other stimulus in the presence of two stimuli (Posner et al., 1980). Similarly, it can also be defined as the tendency for attentional bias, where objects and events associated with one’s experiences and experiences receive greater emphasis (Rosenberg, 2013).

Children’s neuropsychological characteristics are demonstrably related to juvenile offenses and victimization. However, forensic psychology studies on executive functions or attention bias with children are limited in this context. For example, a study by Miller (1997) evaluated the relationship between violent behavior and executive functions of adolescents. Its findings revealed that the group with highly violent behavior obtained lower scores in almost all categories of the Wisconsin Card Sorting Test (WCST) than the control group. In addition, Dalgleish et al. (2001) showed that a study group consisting of children between 9 and 17 diagnosed with post-traumatic stress disorder (PTSD) selectively directed their attention more to stimuli that posed a social danger and avoided stimuli associated with depression; this avoidance decreased with age compared to undiagnosed controls.

Beers and De Bellis (2002) compared 14 adolescents who had experienced sexual or physical maltreatment with 15 healthy adolescents, socio-demographically matched, using the WCST, Stroop test, and Digit Attention Test. The group diagnosed with PTSD due to battery showed significantly decreased executive function and attention performance compared to the healthy control group. In addition, adolescents diagnosed with PTSD showed more impulsivity and distractibility and made more errors in long-term tests measuring attention. Similarly, Malarbi et al. (2017) examined the evidence for cognitive abnormalities in trauma-exposed children with and without PTSD. A total of 1,526 participants from 27 studies were included in the meta-analysis, comprising 412 traumatized children (PTSD unknown), 300 children with PTSD, 323 children without PTSD, and 491 controls. As a result, compared to controls, trauma-exposed children showed cognitive impairments, with PTSD-related deficiencies being the most severe.

Rosser et al. (2005) compared the performance of adolescents with heroin addiction using Towers of Hanoi, a task that measures frontal lobe functions with healthy controls. The findings revealed that adolescents with heroin addiction made more moves while completing the task and completed the task in a shorter time than healthy controls. Furthermore, in a study using the Mind Reading from the Eyes test, which measures the social cognition functions of executive functions, the test performance of children with alcohol-dependent parents was lower than that of children with healthy parents (Hill et al., 2007).

In a study on high-school-age adolescents by Fikke et al. (2011), participants with various psychiatric disorders who exhibited self-destructive behaviors were classified according to the severity of self-destruction and compared with adolescents with healthy executive functions. Adolescents in the group exhibiting high-severity self-destructive behaviors had impaired working memory; adolescents in the group exhibiting low-severity self-destructive behaviors had impaired inhibition control. In addition, a longitudinal study found that children 3–11 years old with poorer self-control had lower economic earnings, poorer health status, and a higher tendency to commit crimes than children with better self-control 30 years later (Moffitt et al., 2011). From a sample of male adolescents between aged 12–18 accused of theft crime, the most frequently observed psychiatric disorders were attention-deficit, hyperactivity, and depressive disorder. Moreover, the scores of the study group were lower than the control group regarding executive function performance (Şenses et al., 2014). Meta-analyzes support the relationship between attention deficit hyperactivity disorder (ADHD) or antisocial behavior between executive functions. Mohr-Jensen and Steinhausen (2016) aimed to review and estimate the risk of arrests, convictions, and incarcerations associated with childhood Attention Deficit/Hyperactivity Disorder (ADHD) in long-term outcome studies. The study included 15,442 individuals from nine unique samples. The results showed a significant association between childhood ADHD and adolescent and adulthood arrests, convictions, and incarcerations. ADHD individuals were younger at the onset of antisocial involvement, increasing the risk of criminal recidivism. The most frequently committed criminal offenses were theft, assault, drug-and weapon-related crimes. Early antisocial behavior problems, childhood maltreatment, sex, and IQ were identified as potential predictors for antisocial outcomes. The findings support a substantial long-term risk associated with ADHD for later antisocial involvement. Gil-Fenoy et al. (2018) conducted a meta-analysis of 33 published articles from 2014 supports the existence of executive alteration in young offenders, which could be influenced by age and test type.

Based on the hypothesis that chronic exposure to maltreatment during childhood may cause impairment in the executive functioning of individuals, Kavanaugh et al. (2017) revealed that maltreated children had a different cognitive control system compared to the control group–specifically, the maltreated group obtained lower performance scores than the control group. Additionally, Ünsal (2018), evaluating two important functions of self-control, inhibition control and working memory, found that the executive functions of adolescents aged 12–17 diagnosed with Internet addiction were significantly lower than those of healthy adolescents. Coenen et al. (2021) explores the role of executive dysfunctions in juvenile delinquency and their integration with self-control in predicting offending. It involved 34 boys in the juvenile justice system and 36 age- and education-matched control boys. Results showed that cool executive functioning was a significant predictor of group, while hot executive functions or self-control were not predictive. Executive functions were not significantly related to self-control scores, suggesting a potential role for cool executive functioning in explaining juvenile delinquency, independent of self-control. The concept of crime is multidimensional and needs to be approached from both the perspective of offenders and victims. For offenders, it is emphasized that the neurobiological basis of aggressive behavior should be examined in terms of variables such as impulsivity, risk-taking, pleasure-seeking, tolerance to frustration, the impact of substance use, working memory, inhibition control, and attention functions (Oquendo and Mann, 2000; Stanford et al., 2005; Cardinal et al., 2006; Yazıcı and Yazıcı, 2010; Krämer et al., 2011; Hagen et al., 2016). Moreover, it is debated that psychiatric disorders like PTSD can also be observed in crime victims (Öztop and Özel-Özcan, 2010) and may cause impairments in the cognitive functions mentioned above (Barrera et al., 2013; Soysal, 2015). When children become the subjects of crime, a concept that affects societies in terms of social, psychological, and economic factors, it is believed that comprehensive research and practices are needed to ensure that past victims do not become future offenders (Sokullu Akıncı, 2011).

Upon examining the literature, various studies have examined reasons or factors that lead children to become involved in or victimized by crime. However, fewer studies focus on child victims of any crime than those dragged into crime, and the issue of child victimization is focused chiefly on child neglect and abuse in the present scholarship. Therefore, this study evaluated the neuropsychological characteristics of children dragged into crime, child victims of crime, and a control group (children without a history of being dragged into crime or victimization) by evaluating their executive function and attention bias. Unfortunately, the relevant literature revealed no studies comparing the executive function skills and attentional biases of children who were dragged into crime, crime victims, and children in the control group using the measurement techniques of this study. In this study, three groups were compared to identify some neuropsychological functions in the background of juvenile delinquency and neuropsychological problems that may occur after victimization or risk factors in becoming a victim of crime. In this context, the following hypotheses were tested.

Hypotheses:

1. The executive functioning of the juvenile offender group is worse than the juvenile victim and the control group.
2. The juvenile offender group tends to show more attention bias toward negative stimuli than the victimized and control groups.
3. The executive functioning of the control group is better than that of the juvenile offender and victimized groups.

## 2. Materials and method

### 2.1. Participants

The number of participants was determined using the G*Power program version 3.1. with a Type-I error rate of 0.05 and a power rate of 80%, the calculated minimum sample size is 93 individuals. However, the total sample size of the study was 85 children, as 10 children dragged into crime and three victim children were excluded from the analysis due to incomplete application. There were no missing values in the analyzed data. The sample of the study group consisted of 60 children between 12 and 18 years old. In addition, 30 participants had a criminal case file opened in Antalya Courthouse for any reason as juvenile offenders and 30 juvenile victims from case files in the courthouse. The control group sample consisted of 25 children between 12–18 who did not have experience of being juvenile offenders or victims in any criminal case file and lived in Antalya province.

The inclusion criteria for the control group were determined as children between 12–18 who could read and write at a comprehensible level, whose mother tongue was Turkish, who did not have intellectual or learning disabilities, who lived in Antalya province, who did not have a history of juvenile offenses or victimization in any criminal case file, whom their parents approved to participate in the study, and who volunteered for the study. The inclusion criteria for the study group were determined as children between 12–18 who could read and write at a comprehensible level, whose mother tongue was Turkish, who did not have intellectual or learning disabilities, who had a criminal case file opened against them for any reason at the Antalya Courthouse and who participated in the case as a child dragged into crime or as a victim, and who volunteered for the study. The children who did not meet these inclusion criteria were excluded from the study and control groups. The types of crimes specified in the criminal case files of the study group are presented in the Table 1.

**Table 1:** Types of crimes.

Types of crimes	Juvenile offender		Juvenile victim		Control group	
	*n*	%	*n*	%	*n*	%
Assault	12	40	11	36.67	0	0
Theft and robbery	9	30	9	30	0	0
Possession of firearms and knives in violation of the law	2	6.67	0	0	0	0
Obscenity	1	3.33	1	3.33	0	0
Property damage	3	10	1	3.33	0	0
Sexual abuse	2	6.67	8	26.67	0	0
Drug-related offenses	1	3.33	0	0	0	0
Children without any type of crime and victimization story	0	0	0	0	0	0
Total	30	100	30	100	25	100

### 2.2. Instruments

After completing the Personal Information Form, the participants were administered the Wisconsin Card Sorting Test (For measuring: Working memory, Abstract thinking, Feature specification, Perseveration, and Conceptualization), the Stroop Test TBAG Form (For measuring: Attention processes, Information processing speed, Inhibition control, and Speed to resist the disruptive effect), the Raven Standard Progressive Matrices Test (For measuring: Analytical evaluation, Problem- solving, Organized thinking, Abstraction, Speed of mental activity, and Visual–spatial perception ability), the Barratt Impulsivity Scale Short Form (For measuring: Motor impulsivity, Attention impulsivity, and Inability to plan) the Istanbul 5 Cube Planning Tower Test (For measuring: Planning skills and Inhibition), and the dot-probe task (For measuring: Attention bias, Impulsivity, and Inhibition), which was created by editing the photographs obtained through International Affective Picture System (IAPS) with the Superlab 6 experiment program.

#### 2.2.1. Personal information form

This form requests the demographic information of the participants. This demographic information includes questions to determine socioeconomic statuses, such as age, gender, education level, place of residence, occupation, and income level. In addition, it includes questions on chronic illness; self-harming behavior; smoking, alcohol, and drug use; running away from home or institutions, a history of crime or victimization and drug use in parents, siblings, or friends; and sociodemographic information of parents.

#### 2.2.2. Wisconsin card sorting test

Heaton (1981) finalized the WCST–initially developed by Berg (1948) to assess conceptualization and abstraction skills (as cited Üney, 2014). The standardization studies in Turkey were conducted by Karakaş (2004). A total of 13 separate scores are calculated in the test. In this study, WCST1 (total number of responses), WCST3 (total number correct), WCST4 (number of categories completed), WCST5 (number of perseverative responses), WCST6 (number of perseverative errors), WCST8 (perseverative error percentage), WCST9 (number of responses used to complete the first category), WCST11 (conceptual level response percentage), WCST12 (failure to sustain setup) scores were calculated. The nature of WCST (Wisconsin Card Sorting Test) does not allow for the calculation of any type of reliability coefficient.

#### 2.2.3. Stroop test TBAG form

The original form of the test was first developed by Stroop (1935) to assess selective attention and response inhibition. The Stroop effect occurs in situations where it is necessary to say the colors of incongruent stimuli printed with a color other than the color it expresses, and it refers to the increased response time as a result of the automatic reading response’s interference effect on color saying (MacLeod, 1992). The TBAG Form of the Stroop Test used in the study was created by combining the original Stroop test and the Victoria Form (Spreen and Strauss, 1991). The standardization and validity-reliability studies in Turkey were conducted by Karakaş et al. (1999). The Stroop Test TBAG Form measures focused attention, the ability to suppress a habitual behavior pattern, the ability to perform an unusual behavior, and the ability to change the perceptual setup under a disruptive effect and in line with changing demands (Karakaş and Doğutepe-Dinçer, 2011). Kılıç et al. (2002) found that the test–retest reliability coefficient ranged between 0.63 and 0.81 on a sample of children aged between 6 and 11 years with a 12-month interval between the tests.

#### 2.2.4. Raven’s standard progressive matrices

The original form of the test was developed by Raven in 1938 to measure analytical evaluation, problem-solving, organized thinking, abstraction, and speed of mental activity, and the test was modified in 1947 and 1956 (O’Leary et al., 1991). RSPM measures Spearman’s “g factor”: general cognitive ability. This factor is related to high-level mental processes such as creativity, speed, diversity in thinking, the ability to improvise and make multi-stage plans when the right solution cannot be reached, and the ability to make thoughts and behaviors more punctual and organized (Karakaş and Doğutepe-Dinçer, 2011). The validity study of the test in Turkey was conducted within the scope of BILNOT-Child Battery. The test comprises five sets (A, B, C, D, E) and 60 items. Each set contains 12 items of increasing difficulty. The items in sets A and B have 6 options, while those in sets C, D, and E have 8 options. While applying the test, the child must comprehend meaningless shapes to determine the feature of the shape that will complete the system of relationships and develop a systematic examination approach (Karakaş and Doğutepe-Dinçer, 2011). In the reliability study conducted by Karakaş and Başar (1993), the correlation coefficient for the total score calculated in the RSPM was found to be 0.79 (*p* < 0.001), and the reliability coefficient for the time scores was 0.64 (*p* < 0.001).

#### 2.2.5. Barratt impulsivity scale short form

The scale was first developed by Barratt (1959) to assess impulsivity and has undergone various revisions over time, and the version used today was developed by Patton et al. (1995). The Barratt Impulsivity Scale Short Form was organized by Spinella (2007). The adaptation of the scale to our culture was conducted by Tamam et al. (2013). The scale consists of 15 items and three sub-dimensions (attention impulsivity, motor impulsivity, and lack of planning). The level of impulsivity is understood from the high total score on the scale, and the higher the score, the higher the level of impulsivity. As a result of the adaptation study determined internal consistencies as Cronbach’s alpha: 0.82 for the total scale, 0.80 for the lack of planning subscale, 0.70 for motor impulsivity and 0.64 for attention impulsivity.

#### 2.2.6. Istanbul 5 cubes planning tower test

This test was developed by Cinan (2015) to assess planning skills inspired by the Hampshire Tree Test and the Tower of London. Tower tasks are also sensitive to executive functions, including the attention group and working memory. Participants access this test through a computer application and try to solve problems using a mouse. The first two stages of the test are called “practice tasks,” with 10 tasks of increasing difficulty. Each stage has a problem the participant must solve using their planning skills. The problem of the tenth stage is the most challenging task in the test. There are different ways for the participant to solve the given problems. However, since there is a “best” solution for each problem, the participant has to follow that solution to succeed in the application. Before each task, participants were given 10 s for planning. The overall reliability coefficient of the test was 0.61. Test–retest reliability scores were found as follows: total score (solving correctly) 0.42, excess movement 0.59, and total solving time 0.47. Due to the test being a cognitive task involving “novelty,” it is expected that the reliability coefficients are not very high.

#### 2.2.7. Dot-probe task

This task was first developed by Posner et al. (1980). Later, adaptation studies were conducted by MacLeod et al. (1986) to assess attentional bias. In the dot-probe task, two words or pictures are shown on a computer screen’s top, bottom, left, and right sides. The pictures used are stimuli with emotional content. Stimulus pairs are neutral-neutral, positive-neutral, and negative-neutral. Two stimulus pictures or words are displayed on the screen simultaneously. The selected words or pictures disappear after a certain period on the screen. After the stimulus disappears, a neutral probe appears at the location of one of these two different stimuli, and the participant is expected to locate this probe as quickly as possible. To locate the probe, the participant must press the appropriate key (e.g., ‘M’ if on the right and ‘X’ if on the left). A short reaction time indicates that the participant is already looking at that location, while a long reaction time indicates that the participant has shifted his/her attention from the other location.

In this study, the Superlab Experiment Program was used to create and implement the dot-probe task and to record the data. The pictures used as negative, positive, and neutral stimuli in the task were selected from the IAPS (Lang et al., 2008). IAPS is an internationally accessible set of emotionally stimulating standard color photographs covering different semantic categories. Previous studies on this subject were reviewed while determining the image pairs to use. Ultimately, 88 neutral visual stimuli, 24 negative visual stimuli, and 24 positive visual stimuli were selected for the task. The pictures selected as negative visual stimuli included images of wild animals, human faces with negative emotional expressions, or images that could threaten physical integrity, such as natural disasters or traffic accidents. The pictures selected as positive emotional stimuli included human faces with positive emotional expressions, cute baby animals, or images of babies and young children; the neutral images included images of objects used in daily life.

Eight trial types were created for each stimulus pair: (1) negative visual stimulus location on the right, dot left, (2) negative visual stimulus location on the right, dot right, (3) negative visual stimulus location on the left, dot right, (4) negative visual stimulus location on the left, dot left, (5) positive visual stimulus location on the right, dot right, (6) positive visual stimulus location on the right, dot left, (7) positive visual stimulus location on the left, dot right, (8) positive visual stimulus location on the left, dot left. All pictures were shown in dimensions of 8.5 cm x 7.5 cm. with a fixation (+) sign between them in the center of the screen in “Courier New” font with a font size of 70 points. The image pairs were presented simultaneously for 1,000 ms, one on the right and one on the left side of the screen. After 1,000 ms, a dot (probe) appeared on the right or left. The participant’s response time to the dot was limited to 1,500 ms. If no response was given within 1,500 ms, it was considered “no response” and incorrect. It takes a maximum of 3,000 ms for the participant to complete a trial.

### 2.3. Procedure

First, permissions from the Ethics Committee of Akdeniz University and the Ministry of Justice, Department of Judicial Support, and Victim’s Rights were obtained. Next, certified application training organized by Neurometrika Medical Medicine Technologies Company was received for the application of the neuropsychological tests–WCST, the TBAG Form of the Stroop Test, and the RSPM Test–before starting the data collection phase.

At the beginning of the applications, the informed consent form was presented to the children in the study and control groups, the latter of whose parents provided consent, and the necessary information about the applications were given. Then, the interventions were carried out individually in a quiet, bright, and distraction-free room. During the interventions, only the researcher and the child were present in the room. A Lenovo V155 81V50010TX model computer was used to implement the Istanbul 5 Cube Planning Tower and dot-probe task.

After completing the Personal Information Form, participants completed the Barratt Impulsivity Scale Short Form and the Raven Standard Progressive Matrices Test individually. Next, the Stroop Test-TBAG Form and Wisconsin Card Sorting Test were administered to the children by the practitioner. Then, computer-based tasks–the Istanbul 5 Cube Planning Tower and the dot-probe task–were presented to the participants. Finally, the participant was instructed to perform the Istanbul 5 Cube Planning Tower task with a mouse.

Finally, the children were directed to the dot-probe task. The child was asked to look at the fixation (+) sign in the middle of the computer screen and react as quickly as possible to the position of the dot that appeared after the pictures. When the child pressed any letter key on the keyboard, the fixation (+) sign remained on the screen for 500 ms. Then, the task began, during which 24 pairs of neutral–neutral stimuli, 20 pairs of positive–neutral stimuli, and 20 pairs of negative–neutral stimuli were presented.

Necessary instructions were given to the children before each intervention. The completion time of all interventions was approximately 75 min.

### 2.4. Statistical analysis

Statistical analyzes were completed using SPSS version 22.0 (Statistical Package for the Social Sciences, SPSS Inc.). The significance value was accepted as *p* < 0.05 at a 95% confidence interval in the analyzes. No missing data were encountered since the applications were carried out one-to-one by the researcher and controlled during the application.

Pearson Chi-Square test and Fisher’s Exact test were used to analyze the categorical data of the cases. For the analysis of continuous data, it was first examined whether the data showed normal distribution using the Shapiro–Wilk test data due to the number of samples. Then, the histogram graphs of the data that did not have a significant value according to the test were also examined, and it was accepted that they showed normal distribution; for the data that had a significant value according to the test, the skewness kurtosis values were examined, and it was accepted that the data between −1.5 and + 1.5 values also showed normal distribution (Tabachnick and Fidell, 2013) and parametric tests were applied in the analysis of these data.

Within the scope of the study, a one-way analysis of variance (ANOVA) was used to determine whether the scores obtained from the applications between the groups of children who were dragged into crime, children who were victims of crime, and children who were not involved in the criminal justice system differed from those with normal distribution. First, the homogeneity of the variances was analyzed with Levene’s test. If the data variances found to have a significant difference between the groups were homogeneous, analyzes on which group or groups differed from each other and in what direction were performed with the Tukey test. In cases where the variances were not homogeneous, the Games Howell test was used to identify the group or groups that differed. The effect size of the difference between the three groups was determined by Eta squared (η2) measurement. Finally, the Kruskal-Wallis H test, a non-parametric test, was used to measure non-normally distributed data. Accordingly, if there was a significant difference between the groups, the Mann–Whitney U test was applied in pairs to determine which group differed.

## 3. Results

### 3.1. Analysis of socio-demographic data

A significant difference was found between the mean age of the juvenile offender group (*M* = 15.97, SD = 1.35) and the mean age of the control group (*M* = 15.00, SD = 1.36) [*F*(2,82) = 3.90, *p* = 0.024, Ƞ2 = 0.08]. No significant difference was found between the other groups in terms of age (*p* > 0.05).

In Table 2, the chi-square analysis results are presented regarding the demographic information of the sample group and the social factors considered significant for children involved in the juvenile justice system (such as parents’ income level or friends’ situation‖). All variables, except for specific characteristics related to parents, significantly differentiate the statuses of children dragged into crime and victims from those in the control group.

**Table 2:** The chi-square analysis of sociodemographic variables.

Sociodemographic variables		Juvenile Offender		Juvenile Victim		Control Group			
		*n*	%	*n*	%	*n*	%	*χ* ^2^	*p*
Sex	Female	7	23.3	11	36.7	14	56	6.22	0.045*
	Male	23	76.7	19	63.3	11	44		
Educational status	Continuing education	15	50	24	80	24	96	15.88	0.000***
	Not continuing education	15	50	6	20	1	4		
Working status	Working	14	46.7	3	10	24	96	18.34	0.000***
	Not working	16	53.3	27	90	1	4		
Self-destructive behavior	Yes	9	30	3	10	0	0	10.42	0.003**
	No	21	70	27	90	25	100		
Escape behavior	Yes	11	36.7	6	20	0	0	11.46	0.003**
	No	19	63.3	24	80	25	100		
Harmful habit cigarette	Yes	18	60	13	43.3	5	20	8.96	0.011*
	No	12	40	17	56.7	20	80		
Harmful habit alcohol	Yes	17	56.7	10	33.4	0	0	20.46	0.000***
	No	13	43.3	20	66.6	25	100		
Harmful habit drugs	Yes	6	20	1	3.4	0	0	7.16	0.016*
	No	14	80	19	96.6	25	100		
Status of parents	Mother–father alive	27	90	28	93.3	25	100	9.31	0.157
	Mother alive–father not	2	6.7	0	0	0	0		
	Father alive–mother not	0	0	2	6.7	0	0		
	Mother–father not alive	1	3.3	0	0	0	0		
Marital status of the parents	Married	22	73.3	19	63.3	17	68	2.94	0.483
	Divorced/living apart	7	23.3	11	36.7	8	32		
	Not alive	1	3.3	0	0	0	0		
Mother’s education level	Illiterate	3	10	0	0	0	0	22.32	0.002**
	Primary school graduate	17	56.7	17	56.7	5	20		
	Secondary school graduate	6	20	6	20	3	12		
	High school graduate	2	6.7	3	10	10	40		
	Graduated from a university	2	6.7	4	13.3	7	28		
Father’s education level	Illiterate	0	0	0	0	0	0	19.58	0.002**
	Primary school graduate	17	56.7	11	36.7	5	20		
	Secondary school graduate	5	16.7	3	10	2	8		
	High school graduate	5	16.7	11	36.7	4	16		
	Graduated from a university	3	10	5	16.7	14	56		
Mother’s income level	Not working	15	50	15	50	8	32	22.72	0.001**
	Less than 5,000 TL	8	26.7	3	10	0	0		
	Between 5,000–7,500 TL	6	20	5	16.7	9	36		
	More than 7,500 TL	0	0	5	16.7	8	32		
Father’s income level	Not working	3	10	1	3.3	0	0	24.97	0.000***
	Less than 5,000 TL	5	16.7	1	3.3	0	0		
	Between 5,000–7,500 TL	11	36.7	15	50	3	12		
	More than 7,500 TL	9	30	9	30	22	88		
Crime/victimization history in mother	Yes	6	20	3	10	1	4	3.502	0.227
	No	24	80	27	90	24	96		
Crime/victimization History in father	Yes	10	33.3	12	40	2	8	7.49	0.024*
	No	20	66.7	18	60	23	92		
Crime/victimization history in siblings	Yes	13	43.3	6	20	1	4	12.05	0.002**
	No	17	56.7	24	80	24	96		
Crime/victimization history in friends	Yes	25	83.3	18	60	2	8	31.99	0.000***
	No	5	16.7	12	40	23	92		
Drug use in mother	Yes	2	6.7	1	3.3	0	0	1.55	0.772
	No	28	93.3	29	96.7	25	100		
Drug use in father	Yes	3	10	0	0	1	4	2.94	0.202
	No	27	90	30	100	24	96		
Drug use in siblings	Yes	4	13.3	3	10	0	0	3.496	0.213
	No	26	86.7	27	90	25	100		
Drug use in friends	Yes	10	33.3	4	13.3	0	0	11.59	0.003**
	No	20	66.7	26	86.7	25	100		

*p < 0.05, **p < 0.01,***p < 0.001.

### 3.2. Analyzes of Barratt impulsivity scale short form

According to the ANOVA conducted to determine the difference between the scores obtained by the children in the juvenile offender, victim, and control groups, the total impulsivity scores obtained by the children showed a statistically significant difference according to the groups they were in [*F*(2,82) = 5.94, *p* = 0.004, Ƞ2 = 0.12]. Per the analyzes conducted to determine which groups differed from each other and in which direction, the impulsivity scores of juvenile offender children (*M* = 31.17, SD = 7.31) were significantly higher than the impulsivity scores of victimized children (*M* = 26.77, SD = 6.24) and the impulsivity scores of children in the control group (*M* = 25.96, SD = 4.23). No significant difference was found between the impulsivity scores of the victimized children and those in the control group (*p* > 0.05).

### 3.3. Analyzes of raven standard progressive matrices test

According to the ANOVA conducted to determine the difference between the scores obtained by the children in the juvenile offender, victim, and control groups, it was found that the scores obtained by the children showed a statistically significant difference according to the groups they were in [*F*(2,82) = 25.70, *p* = 0.000, Ƞ2 = 0.38]. On the one hand, from the analyzes conducted to determine which groups differed from each other and in which direction, the RSPM test scores of the children in the control group (*M* = 45.68, SD = 6.27) were significantly higher than those of the juvenile offender (Mean = 36.53, SD = 5.64) and the scores of the victimized children (*M* = 38.10, SD = 4.69). On the other hand, no significant difference was found between the RSPM scores of children juvenile offenders and victimized children (*p* > 0.05).

### 3.4. Analyzes of Stroop test TBAG form

According to the ANOVA conducted to determine the difference between the STP5_correction scores of the children in the juvenile offender, victim, and control groups, it was found that the scores obtained by the children showed a statistically significant difference according to the groups they were in [*F*(2,82) = 12.46, *p* = 0.000, Ƞ2 = 0.23]. In the analyzes conducted to determine which groups differed from each other and in which direction, it was found that the correction scores of the children in the control group (*M* = 0.76, SD = 0.88) were significantly lower than the correction scores of the juvenile offender (*M* = 2.13, SD = 1.31) and the scores of the victimized children (*M* = 2.00, SD = 1.05). There was no significant difference between the STP5_correction scores of juvenile offenders and victimized children (*p* > 0.05).

According to the analyzes conducted to determine the difference between the STP5_duration, STP5_error, and difference in the 3 scores of the children in the juvenile offender, victimized, and control groups: STP5_duration [*X*^2^(2, *N* = 85) = 18. 49, *p* = 0.000], STP5_error [*X*^2^(2, *N* = 85) = 9.01, *p* = 0.011] and difference [*X*^2^(2, *N* = 85) = 21.95, *p* = 0.000] scores. As a result of the analyzes conducted to determine between which groups there was a significant difference, it was found that the duration scores of the juvenile offender were significantly higher than those of the children in the control group in terms of STP5_duration score (*U* = 135.00, *z* = −4.06, *p* = 0.000). Again, it was found that the duration scores of victimized children were significantly higher than those of children in the control group (*U* = 180.00, *z* = −3.30, *p* = 0.001). In terms of the STP5_error score, it was found that the error scores of the juvenile offender were significantly higher than the children in the control group (*U* = 262.50, z = −2.96, *p* = 0.003). Again, it was found that the error scores of the victimized children were significantly higher than the children in the control group (*U* = 312.50, z = −2.12, *p* = 0.034). The difference 3 scores of the children who were juvenile offenders were significantly higher than the children in the control group (*U* = 117.50, *z* = −4.35, *p* = 0.000). Likewise, the difference 3 scores of victimized children were significantly higher than the scores of children in the control group (*U* = 150.50, *z* = −3.80, *p* = 0.000). No significant difference was found between juvenile offender and victimized children in terms of STP5_duration, STP5_error, and difference 3 scores (*p* > 0.05).

### 3.5. Analyzes of Istanbul 5 cube planning tower test

According to the ANOVA conducted to determine the difference between the I5CPTT scores of the children in the juvenile offender, victim, and control groups, the scores obtained by the children showed a statistically significant difference according to the groups they were in [*F*(2,82) = 51.26, *p* = 0.000, Ƞ2 = 0.55]. Per the analyzes conducted to determine which groups differed from each other and in which direction, the scores of the children in the control group (*M* = 5.48, SD = 1.69) were significantly higher than the scores of the juvenile offender (*M* = 1.7, SD = 1.39) and the scores of the victimized children (*M* = 2.53, SD = 1.22). There was no significant difference between the scores of the juvenile offender and victimized children (*p* > 0.05). According to the ANOVA performed to determine the difference between the excess movement scores of the children in the juvenile offender, victim, and control groups, the excess movement scores obtained by the children showed a statistically significant difference according to the groups they were in [*F*(2,82) = 28.15, *p* = 0.000, Ƞ2 = 0.40]. Per the analyzes conducted to determine which groups differed from each other and in what direction, the excess movement of a juvenile offender in completing the test (*M* = 92.50, SD = 47.72) was significantly higher than the excess movement of victimized children (*M* = 65.37, SD = 28.90) and the excess movement of children in the control group (*M* = 23.92, SD = 12.29). The excess movement of victimized children (*M* = 65.37, SD = 28.90) was significantly higher than that of children in the control group (*M* = 23.92, SD = 12.29).

According to the analyzes conducted to determine the difference between the data related to total solving time and total first action duration of the children in the juvenile offender, victim, and control groups, there was a significant difference between the groups according to the total solving time [*X*^2^(2, *N* = 85) = 41.58, *p* = 0.000] and total first action duration [*X*^2^(2, *N* = 85) = 42.63, *p* = 0.000] scores. From the analyzes conducted to determine between which groups there was a significant difference, the total solving time of the juvenile offender was significantly higher than the victimized children (*U* = 295.00, *z* = −2.29, *p* = 0.022) or those in the control group (*U* = 51.50, *z* = −5.47, *p* = 0.000). In addition, the total solving time of the victimized children was significantly higher than the children in the control group (*U* = 51.50, *z* = −5.47, *p* = 0.000). In terms of total first action duration, the first action duration of the juvenile offender was significantly higher than the children in the control group (*U* = 43.50, *z* = −5.60, *p* = 0.000). The first action duration of the victimized children was significantly higher than the children in the control group (*U* = 44.50, *z* = −5.59, *p* = 0.000).

### 3.6. Analyzes of the Wisconsin card sorting test

According to the ANOVA conducted to determine the difference between the WCST3 scores of the children in the juvenile offender, victim, and control groups, the scores obtained by the children did not show a statistically significant difference according to the groups they were in [*F*(2,82) = 2.44, *p* = 0.094]. According to the ANOVA conducted to determine the difference between the WCST5 scores of the children in the juvenile offender, victim, and control groups, the scores obtained by the children showed a statistically significant difference according to the groups they were in [*F*(2,82) = 11.90, *p* = 0.000, Ƞ2 = 0.22]. In the analyzes conducted to determine which groups differed from each other and in which direction, the WCST5 scores of the juvenile offender (*M* = 28.47, SD = 13.32) were significantly higher than the scores of victimized children (*M* = 19.97, SD = 8.83) and children in the control group (*M* = 14.88, SD = 8.24). There was no significant difference between the scores of victimized children and children in the control group (*p* > 0.05).

According to the ANOVA conducted to determine the difference between the WCST8 scores of juvenile offenders, victims, and children in the control group, the scores obtained by the children showed a statistically significant difference according to the groups they were in [*F*(2,82) = 13.48, *p* = 0.000, Ƞ2 = 0.24]. In the analyzes conducted to determine which groups differed from each other and in which direction, the WCST8 scores of the juvenile offenders (*M* = 21.18, SD = 7.68) were significantly higher than the scores of victimized children (*M* = 15.37, SD = 4.93) and children in the control group (*M* = 12.95, SD = 5.19). There was no significant difference between the scores of the victimized children and the children in the control group (*p* > 0.05). According to the ANOVA conducted to determine the difference in WCST11 scores, the scores obtained by the children showed a statistically significant difference by group [*F*(2,82) = 14.39, *p* = 0.000, Ƞ2 = 0.26]. In the analyzes conducted to determine which groups differed from each other and in which direction, the WCST11 scores of the children in the control group (*M* = 70.12, SD = 13.92) were significantly higher than the scores of the juvenile offender (*M* = 49.25, SD = 14.98) and the scores of the victimized children (*M* = 59.46, SD = 14.14). The WCST11 scores of victimized children (*M* = 59.46 SD = 14.14) were significantly higher than those of juvenile offenders (*M* = 49.25, SD = 14.98).

The non-parametric analyzes were conducted to determine the difference between the WCST1, WCST4, WCST9, and WCST12 data of the children in the juvenile offenders, victims, and members of the control group, with significant differences found in WCST1 [*X*^2^(2, *N* = 85) = 17.43, *p* = 0.000], WCST4 [*X*^2^(2, *N* = 85) = 20.92, *p* = 0.000] and WCST9 [*X*^2^(2, *N* = 85) = 11.36, *p* = 0.003]. There was no significant difference between the groups according to WCST12 [*X*^2^(2, *N* = 85) = 4.16, *p* = 0.125] scores. The WCST1 score of the children in the control group was significantly lower than the children who were juvenile offenders (*U* = 151.00, *z* = −3.98, *p* = 0.000) and victimized children (*U* = 211.00, *z* = −2.83, *p* = 0.005). No significant difference was found between the WCST1 scores of juvenile offenders and victimized children (*p* > 0.05). The WCST4 score of the children in the control group was significantly higher than the children who were juvenile offenders (*U* = 146.50, *z* = −4.33, *p* = 0.000) and victimized children (*U* = 237.50, *z* = −2.85, *p* = 0.004). The WCST4 score of victimized children was significantly higher than juvenile offenders (*U* = 293.00, *z* = −2.45, *p* = 0.014). The WCST9 score of the children in the control group was significantly lower than that of those accused of a crime (*U* = 207.00, *z* = −2.87, *p* = 0.004) and victimized children (*U* = 196.50, *z* = −3.05, *p* = 0.002). No significant difference was found between the WCST9 scores of juvenile offenders and victimized children (*p* > 0.05).

### 3.7. Analysis of the dot-probe Mission

According to the non-parametric analyzes conducted to determine the difference between the total reaction time and correct answer scores of the children in the juvenile offender, victim, and control groups, a significant difference was identified between the groups in total reaction time [*X*^2^(2, *N* = 85) = 6.28, *p* = 0.043] and correct answer scores [*X*^2^(2, *N* = 85) = 19.03, *p* = 0.000]. The total reaction time of the children in the control group was significantly lower than that of the victimized children (*U* = 238.00, *z* = −2.32, *p* = 0.021). There was no significant difference between the total reaction times of juvenile offenders and victimized children and juvenile offenders and children in the control group (*p* > 0.05). The correct answer scores of the children in the control group were significantly higher than the juvenile offender (*U* = 133.50, *z* = −4.19, *p* = 0.000) and victimized children (*U* = 189.50, *z* = −3.23, *p* = 0.001). No significant difference was found between juvenile offenders’ and victimized children’s correct answer scores (*p* > 0.05); Table 3.

**Table 3:** Neuropsychological characteristics and attentional bias.

		Juvenile offender		Juvenile victim		Control group				
		M	SD	M	SD	M	SD	*F/X* ^2^	*p*	Ƞ^2^
Barratt impulsivity scale short form scores		31.17	7.31	26.77	6.24	25.96	4.23	*F* = 5.94	0.004**	0.12
Raven standard progressive matrices test scores		36.5	5.65	38.10	4.69	45.68	6.27	*F* = 25.70	0.000***	0.38
Stroop test TBAG form	STP5_duration	25.75	7.55	23.71	5.25	19.68	2.55	*X*^2^ = 18.49	0.000***	
	STP5_error	0.37	0.61	0.17	0.38	0.00	0.00	*X*^2^ = 9.01	0.011*	
	STP_correction	2.13	1.31	2.00	1.05	0.76	0.88	*F =* 12.46	0.000***	
	Difference3	11.99	6.46	10.67	4.08	7.17	1.68	*X*^2^ = 21.95	000***	
Istanbul 5 cube planning tower test	Total score	1.70	1.39	2.53	1.22	5.48	1.69	*F* = 51.26	0.000***	
	Excess movement	92.50	47.72	65.37	28.90	23.92	12.29	*F* = 28.15	0.000***	
	Total solving time	628.03	224.14	516.13	150.88	328.80	54.40	*X*^2^ *=* 41.58	0.000***	
	Total first action duration	549.97	208.18	457.93	138.11	282.72	46.03	*X*^2^ = 42.63	0.000***	
Wisconsin card sorting test	WCST1	120.60	13.33	114.97	16.95	99.16	21.03	*X*^2^ = 17.43	0.000***	
	WCST3	72.30	8.62	77.13	8.93	74.39	8.89	*F* = 2.44	0.094	
	WCST4	4.27	1.41	5.13	1.11	5.84	0.47	*F =* 12.46	0.000***	
	WCST5	28.47	13.32	19.97	8.83	14.88	8.24	*F =* 11.90	0.000***	
	WCST8	21.18	7.68	15.37	4.93	12.95	5.19	*F =* 13.48	0.000***	
	WCST9	18.67	8.88	18.33	8.06	12.48	3.95	*X*^2^ = 11.36	0.003**	
	WCST11	49.25	14.98	59.46	14.14	70.12	13.92	*F =* 14.38	0.000***	
	WCST12	1.07	1.05	1.13	1.50	0.48	0.65	*X*^2^ = 04.16	0.125	
Correct answer scores and total reaction time for the dot probe task	Correct answer scores	64.97	2.31	65.67	1.52	66.80	1.47	*X*^2^ = 19.03	0.000***	
	Total reaction time (ms.)	178751.73	29254.94	203999.30	73003.56	168801.32	30685.67	*X*^2^ = 6.28	0.043*	
Trial durations for the dot probe task	Trial1	13285.70	1327.86	13190.23	1386.10	11804.88	556.95	*X*^2^ = 24.59	0.000***	
	Trial2	12480.17	963.62	12448.73	1068.06	11830.68	543.66	*X*^2^ = 8.46	0.015*	
	Trial3	13560.07	1520.02	13041.00	1461.08	11825.56	574.81	*X*^2^ = 21.86	0.000***	
	Trial4	12298.83	1039.90	12344.37	1308.83	11782.44	578.92	*X*^2^ = 3.15	0.207	

*p < 0.05, **p < 0.01,***p < 0.001.

According to the non-parametric analyzes conducted to determine the difference between the trial durations of the children in the juvenile offender, victim, and control groups, a significant difference was found between the groups according to Trial1 duration [*X*^2^(2, *N* = 85) = 24.59, *p* = 0.000], Trial2 duration [*X*^2^(2, *N* = 85) = 8.46, *p* = 0.015] and Trial3 duration [*X*^2^(2, *N* = 85) = 21.86, *p* = 0.000]. No significant difference between the groups was found according to Trial4 duration [*X*^2^(2, *N* = 85) = 3.15, *p* = 0.207]. The Trial1 duration of the children in the control group was significantly lower than that for the juvenile offenders (*U* = 113.00, *z* = −4.43, *p* = 0.000) and victims (*U* = 123.50, *z* = −4.25, *p* = 0.000). In terms of Trial2, the duration of the children in the control group was significantly lower than for the juvenile offenders (*U* = 205.00, *z* = −2.87, *p* = 0.004) and victimized children (*U* = 246.00, *z* = −2.18, *p* = 0.029). In terms of Trial3, the duration of the children in the control group was significantly lower than that of juvenile offenders (*U* = 110.00, *z* = −4.48, *p* = 0.000) and victimized children (*U* = 176.00, *z* = −3.36, *p* = 0.001). No significant difference was found between the Trial1, Trial2, and Trial3 durations of juvenile offenders and victimized children (*p* > 0.05).

## 4. Discussion

In recent years, the increasing inclusion of children in the criminal justice system as “juvenile offenders and “juvenile victims” is a severe concern in many respects. For example, when TURKSTAT data are analyzed from 2021, 132,943 children were brought to security units from being dragged into crime, and 207,999 children were brought to security units due to victimization. These numbers increased by approximately 16 and 22% over the preceding year (TURKSTAT, 2022). The first step to prevent this increase is correctly identifying the risk factors.

This study aimed to evaluate and compare the neuropsychological characteristics of children between 12 and 18 years old and involved in or victimized by crime with children without a history of being crime accusations or victimizations in the province of Antalya through their executive functions and attention bias. After obtaining the children’s sociodemographic information, the neuropsychological characteristics of participants were assessed using the tests outlined in the methodology section, including the WCST, the TBAG form of the Stroop test, RSPM, the short form of the Barratt Impulsivity scale, the I5CPTT, and the dot-probe task.”

Upon examining the relevant literature, no study was found to have compared the executive functioning skills and attention biases of children who were dragged into crime, crime victims, and children in the control group using the measurement mentioned above techniques.

### 4.1. Sociodemographic variables

Two types of variables are important in this context: first, the percentage values of the variables that created a significant difference between the delinquent children, victimized children, and the control group, and second, the variables that did not create a difference. According to the results of the analysis of sociodemographic factors considered relevant in the context of children and crime, when examined, many variables, such as age, gender, educational status, substance use, parents’ educational status, and friends’ substance use status, are compatible with the data of the Turkish Statistical Institute on juvenile delinquency and victimization. Thus, the following variables are important for boys–around age 15 most frequently–and children who run away from home or have harmful habits: parents’ education-income levels, friends having a history of crime or victimization, and having harmful habits. These findings coincide with those of the Turkish Statistical Institute and other publications on the subject, showing that the case groups and the control group’s characteristics align with those of the population (TURKSTAT, 2022).

### 4.2. Neuropsychological characteristics and attentional bias

According to the results of the Short-Form Barratt Impulsivity Scale, applied to determine and compare the impulsivity levels of children dragged into crime, victims, and control group, a significant difference was evident between the groups. The findings show that the total impulsivity scores of the children dragged into crime were significantly higher than those of the victimized children in the control group. The concept of impulsivity manifests itself as impatience, carelessness, risk-taking, seeking excitement and pleasure, minimizing the sense of harm, and extroversion (Yazıcı and Yazıcı, 2010). Impulsivity has been associated with aggression (Stanford et al., 2005) and reflects an inability to plan and a low tolerance for inhibition (Oquendo and Mann, 2000). Thus, when children and adolescents with high impulsivity do not receive the support they need to control their impulses, they have difficulty complying with legal and social rules and become prone to crime. In addition, adolescents’ ability to control their impulses is related to their ability to direct their behavior (Soysal, 2015). Children with high impulsivity scores may have decreased ability to direct their behavior and may thus become involved in crime. A study conducted with male adolescents aged 12–18 accused of theft found that children dragged into crime scored significantly higher on the Barratt Impulsivity Scale than the control group (Şenses et al., 2014).

When the performance of the groups in the RSPM test was compared, the children in the control group’s scores were significantly higher than those of the children who were dragged into crime or victimized by it. Accordingly, the control group’s analytical reasoning, problem-solving, organized thinking, and abstraction skills are better than those of children dragged into or victimized by crime who participated in the study. According to the RSPM scores, no significant difference existed between children dragged into crime and victimized children. In the literature, no study compares children involved in criminal proceedings as victims and children who have never been involved in criminal proceedings using RSPM. In this study, the participant’s high motivation to complete the test and the ability to concentrate on the test during the administration of the RSPM test might affect the score obtained. Administering this test to the victimized children before or after a hearing related to the trial in which they were involved may have posed a problem for them to concentrate on the test.

On the other hand, children victimized by crime may have had impairments in their executive functions according to the nature of the crime. In the study, 40% (*n* = 12) of the child victims were found to be victims of sexual abuse. PTSD is seen as the most common mental disorder, with a rate of 30–50% in children who are victims of sexual abuse (Öztop and Özel-Özcan, 2010). Upon examining the literature, studies indicate that PTSD symptoms may be effective on executive function performance. However, it is unknown whether the victimized children in this study developed PTSD and whether they were officially diagnosed. In one study, the impairment in executive functions was attributed to trauma, regardless of a PTSD diagnosis; therefore, similar neurocognitive disorders occurred in all trauma victims with or without a PTSD diagnosis (Barrera et al., 2013).

The performances of the study participants on the TBAG Form of the Stroop Test were evaluated based on the duration, error, and correction scores in the 5th section, which measures resistance to interference, and the difference 3 scores, which have a high correlation with the 5th section (Karakaş et al., 1999). Accordingly, there was a significant difference between the duration, error, and correction scores and the difference 3 scores of the 5th section of the Stroop test. When the difference was examined, the performance of the children dragged into crime and victimized children were worse than those in the control group in all scores. However, the difference between the children dragged into crime and the children victimized by it (in favor of the victimized children) was insignificant. Stroop interference is associated with frontal region functions, such as directing attention to the desired stimulus when there are two stimuli (Freeman and Beck, 2000). Accordingly, the ability of the case groups to direct their attention to the desired stimulus was impaired. This result aligns with the findings of the RSPM test, with similar findings in the literature. Inhibition control is the ability to control one’s attention, behavior, thoughts, and emotions against internal predisposition and external stimuli and to exhibit more appropriate/necessary behavior (Diamond, 2013). In a review study, the most frequently reported and most severe impairment due to adverse treatment in childhood included: impairments in executive functioning, a different cognitive control system compared to the control group, and scoring lower on performance than the control group (Kavanaugh et al., 2017).

In a study conducted with male adolescents between 12 and 18 accused of theft crime, in addition to impulsivity scores, children dragged into crime took significantly longer than the control group to complete the 5th section of the Stroop test (Şenses et al., 2014). This study found that the habit of using drugs significantly differentiated whether the children were in the group of children dragged into crime, victims, or control group. Various studies also indicate that drug use impairs inhibition control skills. Studies evaluating the Stroop test performance of adolescents with substance use found that substance-using participants showed worse Stroop test performance (Cardinal et al., 2006; Hagen et al., 2016). In addition, this study found that the parent income level significantly differentiated whether the children were in the crime, victim, or control group. Upon examining the present scholarship, many studies conducted with children and adolescents examine socioeconomic levels within the scope of attentional skills. According to a study by Stevens et al. (2009), children with low socioeconomic status had deficits in attentional functions such as stopping dominant reactions and filtering information. In line with these findings, the findings regarding the Stroop test are consistent with the literature.

WCST1 (Total number of responses), WCST3 (Total number of correct responses), WCST4 (Number of categories completed), WCST5 (Total number of perseverative responses), WCST8 (Perseverative error percentage), WCST9 (Number of responses used to complete the first category), WCST11 (Conceptual level response percentage) and WCST12 (Failure to sustain the setup) scores of WCST were analyzed to examine the working memory, abstract thinking, change of setup and mental flexibility skills of the children in the case and control groups. Accordingly, no significant difference between the groups regarding WCST3 and WCST12 scores was found. However, there was a significant difference between the groups in terms of WCST1, WCST4, WCST5, WCST8, WCST9, and WCST11 scores; children dragged into crime scored significantly higher than the control group in the total number of responses (WCST1), the number of perseverative responses (WCST5), percentage of perseverative errors (WCST8), number of responses used to complete the first category. The control group scored significantly higher than the children dragged into crime in the number of completed categories (WCST4) and conceptual level response percentage (WCST11) scores.

Studies report a robust correlation between executive functions and working memory capacity (Mccabe et al., 2010). Furthermore, working memory capacity can retain incoming information and deactivate unnecessary information of fluent intelligence (Engle, 2002), as working memory and inhibition control support each other (Diamond, 2013). Accordingly, children dragged into crime show impaired working memory compared to children in the other groups. There is also an opinion that WCST performance provides information about learning rules (Perrine, 1993). Therefore, children dragged into crime show difficulty obeying and learning social and legal rules. The victimized children in this study had significantly lower scores in the total number of responses (WCST1) and the number of categories completed (WCST4) scores compared to the control group; however, they scored significantly higher in the number of responses used to complete the first category (WCST9) score compared to the control group.

In a study conducted with children diagnosed with PTSD and matched healthy control group children, it was found that children in the case group performed significantly lower than the control group regarding the completed category (WCST4) and the number of perseverative errors (WCST6) scores (Beers and De Bellis, 2002). Although it is unknown whether the victimized children in this study were diagnosed with PTSD, considering the finding that trauma itself causes impairment in executive functions (Barrera et al., 2013), the findings of Beers and De Bellis’ study are consistent with these findings. In addition, this study found that the number of perseverative responses (WCST5) and percentage of perseverative errors (WCST8) of children dragged into crime was significantly higher than the control group. Unlike the Stroop test, inhibition in the WCST is the participant’s perseveration, persistently continuing the behavior despite verbal feedback (Karakaş, 2004). A study found that children with high WCST perseveration scores had a lessened ability to control their aggressive behaviors. This finding explained that children with high perseveration had difficulty suppressing inappropriate behaviors and were insufficient in preventing violent behaviors (Krämer et al., 2011). Thus, the findings in the literature are consistent with this study.

Cinan (2015) developed a new test inspired by the Tower of London and Hampshire Tree Test to assess planning skills. When the literature was examined, no study was found where children dragged into crime and children victimized by crime were evaluated with I5CPTT. Therefore, this study analyzed the data belonging to the groups regarding the score, excess movement, total solving time, and total first action duration of the I5CPTT. There was a significant difference in all dimensions when the findings were analyzed. Accordingly, the control group’s scores were significantly higher than those of the children dragged into crime and the victimized children. Furthermore, excess movement, total solving time, and total first action duration were significantly higher in the victimized children than in the control group and significantly higher among the children dragged into crime than the victimized children. Accordingly, both groups of children victimized by crime and children dragged into committing it seem to possess impaired planning skills. This impairment is highest for children accused of crimes.

In this study, children dragged into crime and children victimized by it also had impaired planning and problem-solving skills. When the tower tests are analyzed, the planning skill covers a limited timeline, while the problem-solving skill covers a broad timeline, including past, future, and present (Tunstall, 1999). In a study by Rosser et al. (2005), the performance of adolescents with heroin addiction at Towers of Hanoi—a task measuring frontal lobe functions–was compared with the healthy control group. According to their findings, adolescents with heroin addiction made more moves while completing the tasks but completed them in a shorter time than healthy controls. The fact that individuals with substance addiction completed the test in a shorter time contradicts the finding in this study—however, this disparity may be due to this study’s changes of less time and less movement in the instructions for the Towers of Hanoi test. According to the instructions in the I5CPTT, only the information that the participants should make fewer movements is presented. In addition, tower tests that measure planning skills present a unique conundrum: there is a strong tendency to make moves that seem close to the target, but that, if done, will increase the number of moves and prevent the correct solution from being reached. Thus, it may be necessary to inhibit these moves and make a distancing move for a successful outcome, so evaluations of “key problem-solving movements” also reveal the inhibition function in tower tests (Cinan, 2015). The impairment in planning and problem-solving skills of children dragged into crime and victimized in this study predicted impairment in inhibition functions. In this respect, the I5CPTT findings are consistent with the Stroop test findings.

The dot-probe task with positive and negative picture stimuli was applied to evaluate the attentional biases of children who were dragged into crime, children who were victims of crime, and children in the control group. Accordingly, there was a significant difference between the groups according to total reaction time and correct answer scores. The total reaction time of the children in the control group was significantly lower than the victimized children, and the correct answer scores were significantly higher than the children dragged into crime and the victimized children. Accordingly, there is a differentiation in the cognitive functions of victimized children compared to the control group. Regarding correct answer scores, when the literature was examined, incorrect answers in the task were associated with decreased attention and concentration and excessive impulsive response styles (Eggers et al., 2013). This finding is consistent with the finding in this study, and thus, the lower correct answers of children dragged into crime and victimized children are associated with impaired attention and concentration and more use of impulsive response styles by these children.

In addition, within the scope of the dot-probe task, the duration of trial1 (position of the negative visual stimulus on the right, dot on the left), trial2 (position of the negative visual stimulus on the right, dot on the right), trial3 (position of the negative visual stimulus on the left, dot on the right) and trial4 (position of the negative visual stimulus on the left, dot on the left) were examined. Accordingly, there was a significant difference between the groups regarding the trial1, trial2, and trial3 durations. Furthermore, the trial1, trial2, and trial3 durations of the control group were significantly lower than those of the children dragged into crime and victimized children. However, no significant difference was found between the groups regarding trial4 duration.

Upon comparing the two trials where the dot and the negative stimulus were in the same position between the groups, the difference in the negative visual stimulus position on the right and the dot on the right condition (trial2 duration) and lack of difference in the negative visual stimulus position on the left and the dot on the left condition (trial4 duration) were thought to be related to the participant’s dominant right- or left-handedness. In the dot-probe task, participants must inhibit the emotional stimulus–the negative visual stimulus–continue the task. When the position of the dot and the position of the negative visual stimulus were reversed, the reaction times of children dragged into crime and victimized children were significantly longer than those of children in the control group. Thus, the ability of children dragged into crime and victimized children to continue the task by inhibiting the negative emotional stimulus appears lower than that of the children in the control group. Dalgleish et al. (2001) showed that children aged 9–17 diagnosed with PTSD selectively directed their attention more to socially dangerous stimuli than undiagnosed controls while they avoided depression-related stimuli; this avoidance decreased with age.

## 5. Limitations

The small number of participants stands out as a significant limitation of this study; nevertheless, because the practices in this study were intensive, took a long time (minimum 75 min), and data were collected from a particular group, such a limitation was necessary due to resources. Another limitation is the unequal gender distribution in the groups. As most of the children dragged into crime were male, it was challenging to establish a gender balance between the groups in the study. And since the data was collected for children who were already applying to the judicial system in a certain period, it was also difficult to control for other socio-demographic and crime related variables (age, types of crime). Furthermore, the measurement tools were applied to the children dragged into crime and victimized in the legal environment, but the control group received them in an ordinary place outside the courthouse. This method was applied since it was impossible to interview children accused of or victimized by crime anywhere other than the courthouse. Finally, in addition to these limitations, the fact that this study was conducted in groups with complex data collection and neuropsychological measurements may be a guide for other studies.

## 6. Conclusion

This study evaluated whether children dragged into crime differed concerning executive functioning and attentional bias compared to victimized children and children not involved in any forensic incident. The Barratt Impulsivity Scale scores of the children who became involved in crimes were significantly higher than the victimized children and the children in the control group. According to the scores of Raven Progressive Matrices Test, Wisconsin Card Sorting Test, Stroop Test TBAG Form, and the Istanbul 5 Cube Planning Test, the control group performed significantly better than the children who were dragged into crime and victimized children. In addition, according to the dot-probe task performances, children in the control group exhibited less attention bias to negative stimuli than children who were dragged into crime and victimized children.

Finally, the executive function skills of the control group were better than those of the victimized children or those accused of criminal behavior. The number of child delinquency and victimization incidences increases daily in our country. The findings supported by relevant meta-analytical studies (e.g., Mohr-Jensen and Steinhausen, 2016; Malarbi et al., 2017; Gil-Fenoy et al., 2018) highlight the importance of applying theoretical knowledge per the cultural and judicial rules. Understanding the neuropsychological functions of children dragged into crime can prevent them from getting involved again and support psychosocial rehabilitation programs. In terms of victimized children, understanding the effect of victimization on children’s cognitive processes can be beneficial for developing programs that will contribute to the post-traumatic growth of victimized children and prevent them from being victimized again. In conclusion, in this study, it is vital to examine the neuropsychological characteristics and attention biases of victimized and victimized children by comparing them with the control group to provide ideas for similar future studies. As in other research, this study is constrained by the abovementioned limitations; more comprehensive studies may address these limitations.

## Data availability statement

The raw data supporting the conclusions of this article will be made available by the authors, without undue reservation.

## Ethics statement

The studies involving humans were approved by Akdeniz University Ethics Committee. The studies were conducted in accordance with the local legislation and institutional requirements. Written informed consent for participation in this study was provided by the participants’ legal guardians/next of kin.

## Author contributions

All authors listed have made a substantial, direct, and intellectual contribution to the work and approved it for publication.

## Conflict of interest

The authors declare that the research was conducted in the absence of any commercial or financial relationships that could be construed as a potential conflict of interest.

## Publisher’s note

All claims expressed in this article are solely those of the authors and do not necessarily represent those of their affiliated organizations, or those of the publisher, the editors and the reviewers. Any product that may be evaluated in this article, or claim that may be made by its manufacturer, is not guaranteed or endorsed by the publisher.
